# Radical-Assisted Formation of Pd Single Atoms or Nanoclusters on Biochar

**DOI:** 10.3389/fchem.2020.598352

**Published:** 2020-11-30

**Authors:** Chong Xiang, Qingya Liu, Lei Shi, Zhenyu Liu

**Affiliations:** State Key Laboratory of Chemical Resource Engineering, Beijing University of Chemical Technology, Beijing, China

**Keywords:** radical, biochar, single atom, nanocluster, Pd

## Abstract

Supported single atom or nanocluster catalysts have been widely studied due to their excellent catalytic properties. Many methods to prepare such catalysts start with constructing defects on supports, and the main focus is to improve dispersion and stability of the active sites. This paper for the first time reports a radical-assisted method to prepare single atom or nanocluster Pd on a biochar. The char was prepared by pyrolyzing walnut shell at 600°C under N_2_, and Pd was loaded on the char by impregnating with palladium acetate in toluene under an oxygen-free atmosphere. It is found that there are three types of radicals in the fresh char (F-Char-600), two of them may adsorb/bond with O_2_ or Pd^2+^ resulting in decreases in the char's radical concentration. The Pd on F-Char-600 for 24 h impregnation are single atoms (0.1–0.3 nm, 2%) and nanoclusters (0.3–1.2 nm, 98%), which grow larger (0.3–4 nm, 100%) for 84 h impregnation. The Pd on N_2_ purged O_2_-adsorbed-char (N-O-Char-600) is much larger in size. The bond between Pd and char is probably C–Pd in F-Char-600 or C–O–Pd in N-O-Char-600.

## Introduction

Catalysts with highly dispersed nanoscale active components on various supports have been studied and used (Huang et al., 2012; Wang et al., 2013; Li et al., 2014; Shi et al., 2014). As the size of active components decreases, their free energy and activity increase, so does their mobility on supports, leading to easy agglomeration and deactivation (Yang et al., 2013), especially when the active component size is down to the single atom level. Many attempts were made to form highly dispersed stable active components on supports (Qiao et al., 2011).

The nature of catalyst supports plays an important role in the formation and stability of active component sites. For single atom catalysts, the supports studied include metal (Georgios et al., 2012), metal oxide (Lin et al., 2012), molecular sieve (Lu et al., 2012), metal organic framework (MOF) (Zhang et al., 2016), and graphene (Wang et al., 2018). These supports contain defects that interact with and stabilize single atoms (Lin et al., 2012; Zhang et al., 2016; Wang et al., 2018). Biochar is also a catalyst support and has been used in chemical preparation (Ormsby et al., 2012), biofuel production (Nieva Lobos et al., 2016), and pollutant control (Cha et al., 2010). The formation of defect sites on its surface and consequently the single atom active components are also of great interest. However, the methods reported on preparing biochars through pyrolysis (Shen and Yoshikawa, 2014) and supporting active components by impregnation (Wang et al., 2014; Nieva Lobos et al., 2016) and sol–gel (Li et al., 2007) resulted mainly in large active component sizes, 1.9–38 nm, for example, without single atom sites.

The supported single atoms have been characterized by high angle annular dark field aberration-corrected scanning transmission electron microscope (HAADF-STEM). Their image intensity was found to be proportional to the square of atomic number (Z^2^). The image also tells the detailed location of a single atom in support structure and the statistical size distribution of the active sites. For instance, Qiao et al. embedded Pt atoms on an iron oxide support and showed by HAADF-STEM that only Pt single atoms are present (Qiao et al., 2011). Yan et al. selectively deoxidized an oxidized graphene to form active hydroxyl sites on its surface and then replaced the hydroxyl's H atoms by Pd atoms through atomic layer deposition to form C–O–Pd linkage that resulted in nano Pd clusters of <1 nm in size and Pd loadings of 0.01–1.70 wt.% (Yan et al., 2015). Bulushev et al. loaded Cu on a N-doped porous carbon network and showed by HAADF-STEM that the doped pyridine N reduced Cu agglomeration through Cu–N coordination, resulting in a small number of single Cu atom sites (Bulushev et al., 2017). Wang et al. blasted holes on the graphene surface by high-energy atoms or ions to generate unpaired electrons and then sputtered various metals (M, such as Pt, Co, and In) into these holes to form single atoms through C–M linkage as evidenced by high-resolution transmission electron microscopy (Wang et al., 2012). Apparently, the formation of single atoms and their linkage to the supports depend on the chemical state of defects in supports. For carbon supported single atom catalysts, the linkages may be C–O–M, C–N–M, and C–M.

It was reported that coal chars from pyrolysis in the temperature range of 300–750°C contain radicals and their concentration maximizes at 600°C (Seehra and Ghosh, 1988; Cheng et al., 2020; Xiang et al., 2020). Some of the radicals adsorb oxygen strongly and irreversibly, whereas some other radicals adsorb oxygen weakly and reversibly (Xiang et al., 2020). Since an oxygen molecule contains two unpaired electrons, it tends to bond with the unpaired electrons on the char surface. This phenomenon implies that metal cations that lack one or more electrons may strongly bond with or anchored at the biochar's radical sites in a metal cation-containing solution. If this is the case, biochar supported single atom catalysts can be prepared by utilizing the biochar radicals. This type of work, however, has not been reported.

## Method

In this work, walnut shell chars were prepared from pyrolysis at a heating rate of 5°C/min to 600 or 850°C as detailed in the Supplementary Material. The whole process was under a flow of Ar (0.99999 purity) at 100 ml/min. The chars were discharged and sieved to <0.25 mm in size under a N_2_ atmosphere in a glovebox to yield the fresh chars F-Char-600 and F-Char-850, respectively. Some of the fresh chars were fully exposed to O_2_ at room temperature to yield the oxygen-exposed chars, termed as O-Char-600 and O-Char-850, respectively. The O-Chars were then subjected to N_2_ purging at room temperature to yield N-O-Char-600 and N-O-Char-850, respectively.

These chars were impregnated with a solution containing palladium acetate and toluene (termed Pd(Ac)_2_/toluene) or tetrahydrofuran (termed Pd(Ac)_2_/THF) under nitrogen for F-Char-600 and F-Char-850 or in a parafilm covered beaker in air for N-O-Char-600 and N-O-Char-850. The chars were also characterized by the ultimate and proximate analyses, electron spin resonance (ESR) for radical concentration *C*_R_, HAADF-STEM, and inductively coupled plasma atomic emission spectroscopy (ICP-AES) for Pd loadings. Details are shown in the Supplementary Material.

## Results and Discussion

**Scheme 1:**
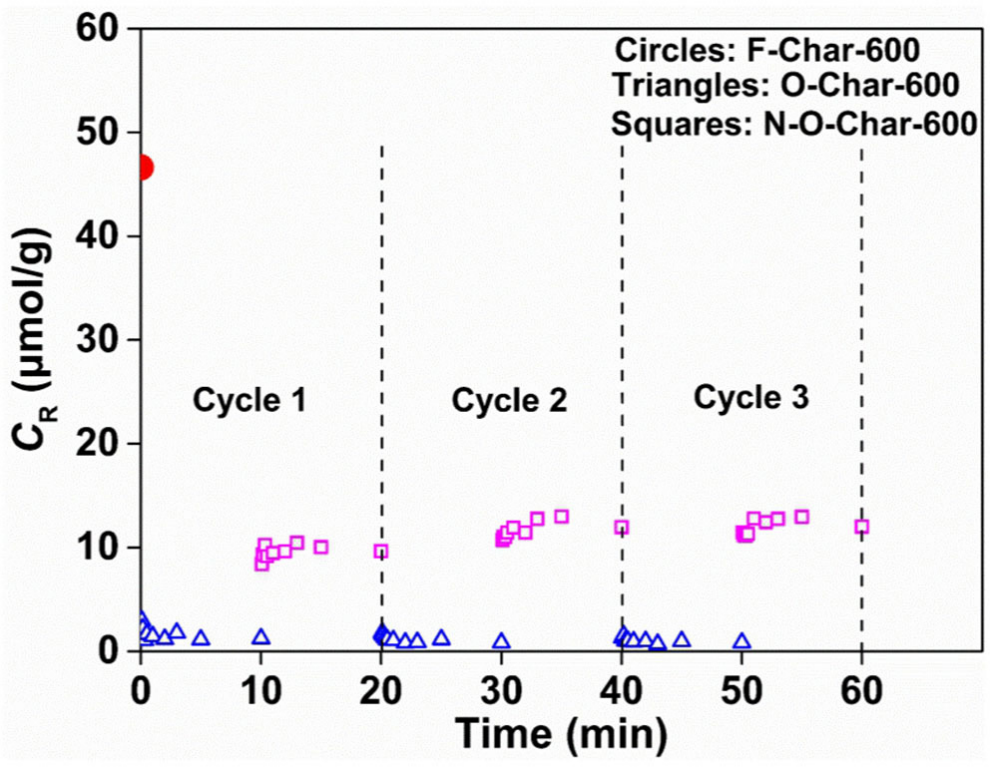
C_R_ change of Char-600 during O_2_ adsorption and N_2_ purging.

Scheme 1 shows the *C*_R_ change of Char-600 during O_2_ exposure and then N_2_ purging. It is seen that the *C*_R_ of F-Char-600 is 46.6 μmol/g (the circle at 0 min). It decreases rapidly to 1.0 μmol/g in O_2_ (the triangles) to form O-Char-600 and then increases to 9.5 μmol/g in N_2_ purging (the squares) to form N-O-Char-600. This trend of *C*_R_ is confirmed by the second and third O_2_-adsorption-and-then-N_2_-purging cycles, indicating a high reliability of the measurement. These data suggest the presence of three types of radical sites in F-Char-600. One type is the strong radical sites that strongly and irreversibly adsorb/bond O_2_ at room temperature, i.e., the adsorbed/bonded O_2_ cannot be purged off by N_2_, and its concentration is about 37.1 μmol/g (46.6–9.5 μmol/g). Another type is the weak radical sites that can weakly and reversibly adsorb/bond O_2_, i.e., the adsorbed/bonded O_2_ can be removed by N_2_ purging, and its concentration is ~8.5 μmol/g (9.5–1.0 μmol/g). The third type is the enclosed radical sites that are confined in the char structure and not able to contact O_2_, and its concentration is about 1.0 μmol/g. This behavior agrees with that of corncob chars (Xiang et al., 2020).

As hypothesized earlier, the radicals on the char surface may bond electron-deficient metal cations, such as Pd^2+^. The amount of Pd^2+^ cations bonded on the strong radical sites would be 18.6 μmol/g-char when a Pd^2+^ cation links two radical sites or 37.1 μmol/g-char when a Pd^2+^ cation links one radical site. Similarly, the amounts of Pd^2+^ cations bonded on the weak radical sites would be 4.3 or 8.5 μmol/g-char when a Pd^2+^ cation links two or one radical site(s), respectively. Therefore, the minimum amount of Pd^2+^ required to bond the strong and weak radical sites on F-Char-600 is approximately 22.9 (18.6 + 4.3) μmol/g-char, corresponding to 2.43 mg Pd/g-char. To avoid agglomeration of Pd particles on the char surface and clearly show the small size of Pd particles, 3.12 mg palladium acetate, corresponding to 1.48 mg Pd, about 60% of the minimum Pd loading was used to prepare a toluene solution (Pd(Ac)_2_/toluene) for impregnation.

**Scheme 2:**
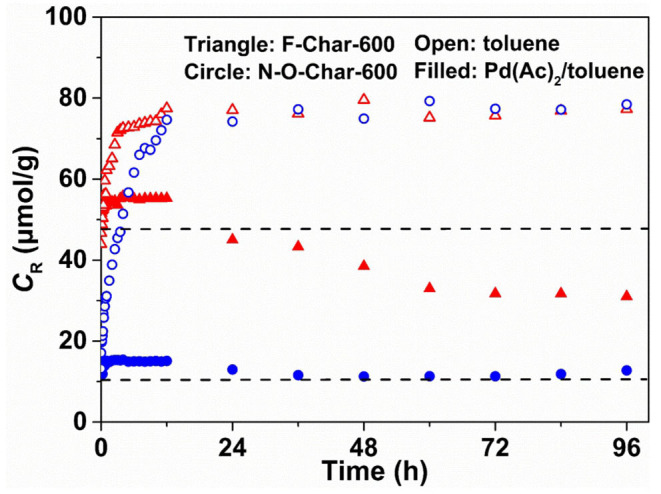
C_R_ change of Char-600 in Pd(Ac)_2_/toluene and toluene.

Scheme 2 shows the *C*_R_ during impregnation of F-Char-600 and N-O-Char-600 by Pd(Ac)_2_/toluene or toluene. The dashed lines are the *C*_R_ of chars alone, 46.6 and 9.5 μmol/g, respectively. It is seen that the *C*_R_ of F-Char-600 in Pd(Ac)_2_/toluene (the filled triangles) increases initially to 55.8 μmol/g, then decreases to 45.0 μmol/g for 24 h, slightly lower than the *C*_R_ of char, and stabilizes at 31.7 μmol/g for 72 h. The initial increase of *C*_R_ is attributed to tar removal from the char because tar contains radicals (He et al., 2014; Wu et al., 2017) that may couple loosely with the char radicals. This hypothesis agrees with the high volatile content of F-Char-600, 26.7 wt.% in Supplementary Table 1, and is consistent with the *C*_R_ behavior of F-Char-600 in toluene (the open triangles) that increases monotonically to 77.5 μmol/g for 12 h. Clearly the trend of *C*_R_ in Pd(Ac)_2_/toluene impregnation can be attributed to two counter effects, the removal of loosely coupled tar radicals from the char radicals by toluene that increases *C*_R_ and the coupling of Pd^2+^ cations with the char radicals that decreases *C*_R_. The former occurred mainly for 4 h, whereas the latter took place mainly for 72 h. Therefore, the Pd loading in F-Char-600 is about 45.8 μmol/g considering the toluene effect or 14.9 μmol/g excluding the toluene effect.

Scheme 2 also shows that the *C*_R_ of N-O-Char-600 in Pd(Ac)_2_/toluene (the filled circles) increases initially and then decreases to a stable value close to the initial *C*_R_, whereas the *C*_R_ of N-O-Char-600 in toluene (the open circles) increases monotonically to values similar those of F-Char-600 in toluene for 12 h. These behaviors suggest that toluene removes not only the tars but also the bonded O_2_ from the N-O-Char-600. If this is the case, it suggests that the amounts of Pd^2+^ cations bonded to the radical sites on the surface of N-O-Char-600 are about 64.2 μmol/g considering the toluene effect, about 1.4 times that on F-Char-600, or about 0 μmol/g excluding the toluene effect.

The effect of tar removal by toluene on *C*_R_ is confirmed by the char impregnation with Pd(Ac)_2_/THF or THF (Scheme S1), during which the trends of *C*_R_ are similar to those in Scheme 2.

**Scheme 3:**
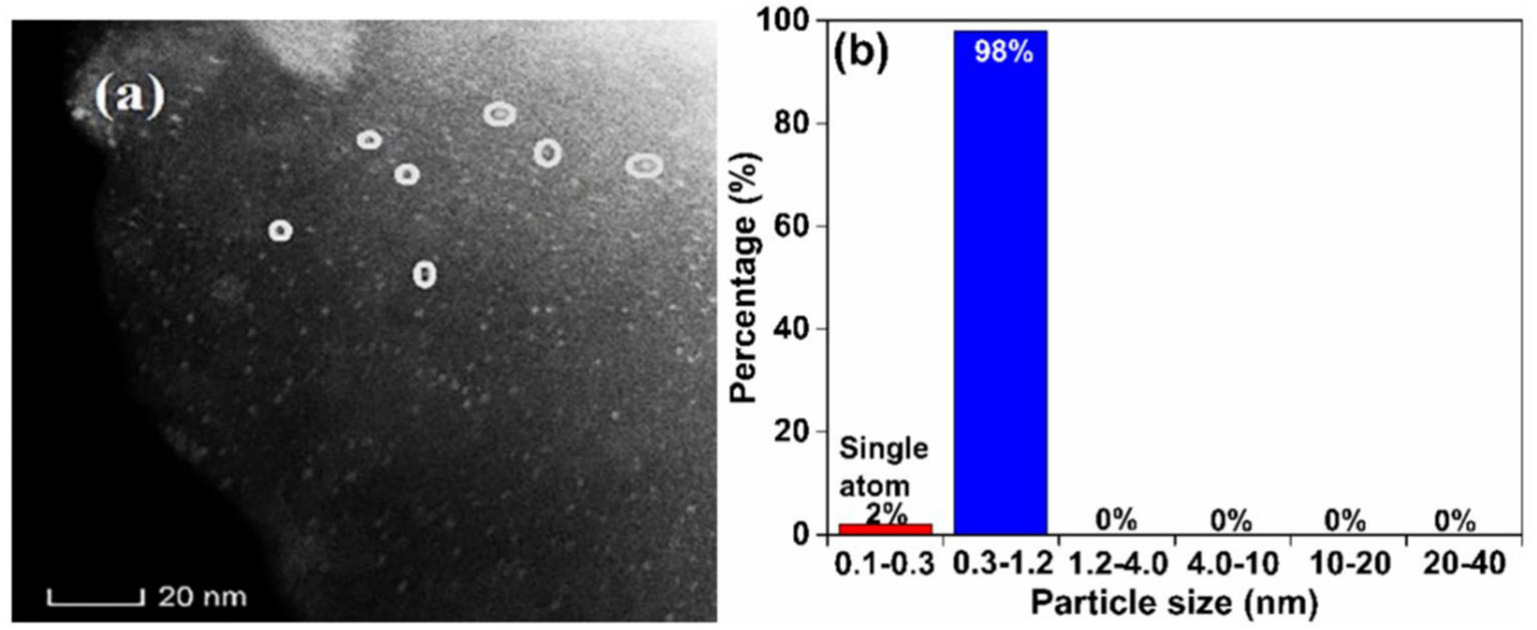
A HAADF-STEM image **(a)** and corresponding Pd size distribution **(b)** of F-Char-600 impregnated with Pd(Ac)_2_/toluen**e** for 24 h.

**Scheme 4:**
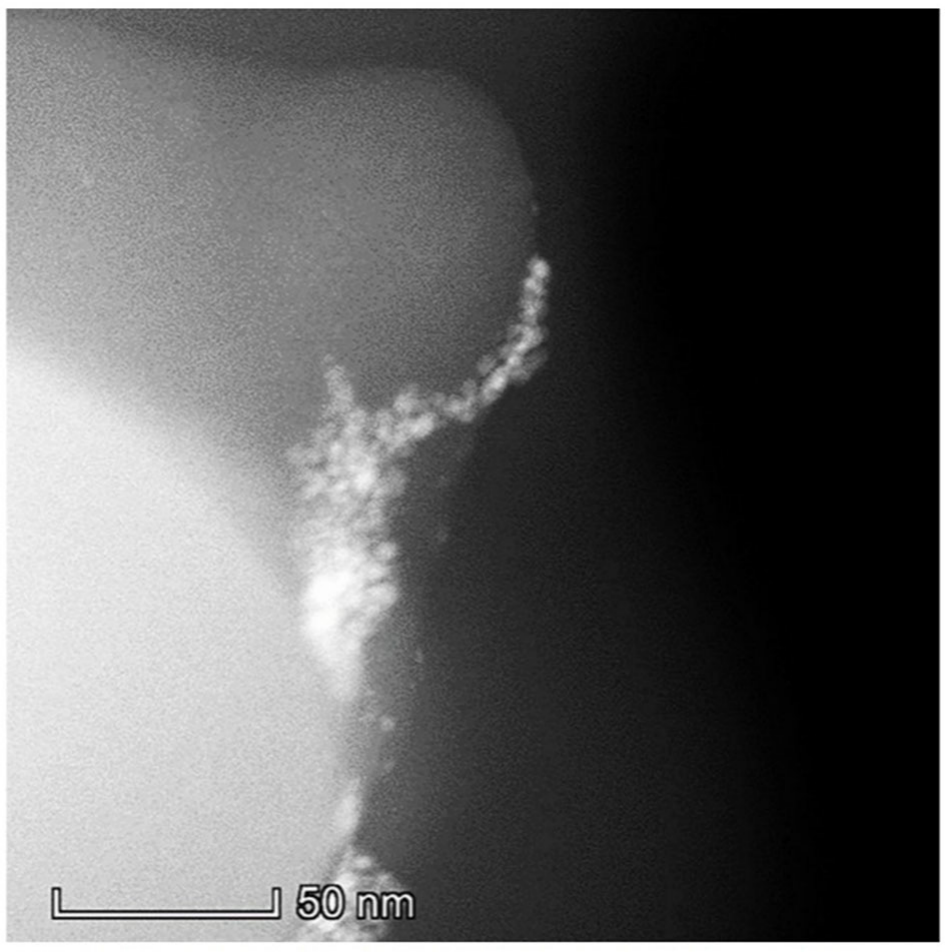
A HAADF-STEM image of N-O-Char-600 impregnated with Pd(Ac)2/toluene for 24 h.

Scheme 3 shows a HAADF-STEM image (a, 100% contrast) and the corresponding Pd size distribution (b) of F-Char-600 impregnated with Pd(Ac)_2_/toluene for 24 h. Clearly, there are many bright spots (marked with the circles) on the char surface (the gray background). The single Pd atoms (0.1–0.3 nm in size) account for about 2 wt.% Pd, whereas the rest are Pd nanoclusters of 0.3–1.2 nm in size. Scheme 4 shows a HAADF-STEM image of N-O-Char-600 impregnated with the Pd(Ac)_2_/toluene also for 24 h (100% contrast). Apparently, only large agglomerated Pd particles are visible on the char surface. These behaviors suggest that the char radicals play an important role in bonding Pd^2+^ cations and preventing them from agglomeration, which however also indicates that the solvent effect is more complex than we had proposed. It is possible that the radicals recovered by the solvents through tar removal are mainly the weak sites, whereas the oxygen bonded strong radical sites on N-O-Char-600 are not recovered by the solvents.

The effect of char radicals on Pd particle size is further elucidated by the HAADF-STEM images of F-Char-600 (Scheme S2a) and N-O-Char-600 (Scheme S2b) impregnated with the Pd(Ac)_2_/toluene for 84 h (100% contrast), as well as the Pd size distribution of F-Char-600 (Scheme S2c). The larger Pd particle size than that in Scheme 3b indicates the growth of Pd particles with impregnation time.

The ICP-AES results (Supplementary Table 2) show that the Pd impregnated F-Char-600 contains 0.017 or 0.061 wt.% Pd, whereas the Pd impregnated N-O-Char-600 contains 0.024 and 0.075 wt.% Pd for 24 or 84 h impregnation, respectively. The trends of these data agree with the HAADF-STEM results, i.e., the Pd size on F-Char-600 is smaller than that on N-O-Char-600, and a longer impregnation time results in a higher Pd content. The Pd loadings are similar to those of single atom catalysts shown in the Supplementary Material.

The effect of char radicals on Pd particle size is further supported by Scheme S3 (100% contrast), where F-Char-850 and N-O-Char-850 contain no radicals, and the Pd particles formed in impregnation with Pd(Ac)_2_/toluene for 84 h are >10 nm.

It is possible that the Pd supported on F-Char-600 forms the C–Pd linkage that is stable and maintains small Pd sizes, whereas the Pd on N-O-Char-600 forms C–O–Pd linkage that is less stable, and the Pd particles tend to migrate and agglomerate on the char surface. Since F-Char-850 and N-O-Char-850 do not have radicals, the Pd supported on their surface does not form C–Pd linkage and tends to migrate on the surface to form large Pd agglomerates.

In conclusion, the radicals on biochar surface are crucial to anchor single Pd atoms or Pd nanoclusters, which is a promising new method to prepare highly active catalysts and the method can be extended to other metals. The method should be further optimized, and the mechanism, the state of the radicals, and the valence of Pd single atoms should be further studied.

## Data Availability Statement

The original contributions presented in the study are included in the article/Supplementary Material, further inquiries can be directed to the corresponding author/s.

## Author Contributions

ZL: conceptualization, methodology, data curation, writing review, and editing. CX: visualization, investigation, and writing original draft preparation. LS: resources and project administration. QL: supervision, discussion, and funding acquisition. All authors have read and agreed to the published version of the manuscript.

## Conflict of Interest

The authors declare that the research was conducted in the absence of any commercial or financial relationships that could be construed as a potential conflict of interest.

## References

[B1] Bulushev D. Chuvilin A. Sobolev V. Stolyarova S. Shubin Y. Asanov I. (2017). Copper on carbon materials: stabilization by nitrogen doping. J. Mater. Chem. A 5, 10574–10583. 10.1039/C7TA02282D

[B2] Cha J. Choi J. Ko J. Park Y. Park S. Jeong K. (2010). The low-temperature SCR of NO over rice straw and sewage sludge derived char. Chem. Eng. J. 156, 321–327. 10.1016/j.cej.2009.10.027

[B3] Cheng X. Shi L. Liu Q. Liu Z. (2020). Heat effects of pyrolysis of 15 acid washed coals in a DSC/TGA-MS system. Fuel 268:117325 10.1016/j.fuel.2020.117325

[B4] Georgios K. Matthew B. April D. Emily A. Timothy J. Ashleigh E.. (2012). Isolated metal atom geometries as a strategy for selective heterogeneous hydrogenations. Science 335, 1209–1212. 10.1126/science.1215864 22403387

[B5] He W. Liu Q. Shi L. Liu Z. Ci D. Lievens C.. (2014). Understanding the stability of pyrolysis tars from biomass in a view point of free radicals. Bioresour. Technol. 156, 372–375. 10.1016/j.biortech.2014.01.063 24507874

[B6] Huang Z. Gu X. Cao Q. Hu P. Hao J. Li J.. (2012). Catalytically active single-atom sites fabricated from silver particles. Angew. Chem. 124, 4274–4279. 10.1002/ange.201109065 22419271

[B7] Li X. Yuan Z. He S. (2014). CO oxidation promoted by gold atoms supported on titanium oxide cluster anions. J. Am. Chem. Soc. 136, 3617–3623. 10.1021/ja412608b 24528209

[B8] Li Y. Zhang S. Yu Q. Yin W. (2007). The effects of activated carbon supports on the structure and properties of TiO_2_ nanoparticles prepared by a sol–gel method. Appl. Surf. Sci. 253, 9254–9258. 10.1016/j.apsusc.2007.05.057

[B9] Lin J. Qiao B. Liu J. Huang Y. Wang A. Li L.. (2012). Design of a highly active Ir/Fe(OH)_x_ catalyst: versatile application of Pt-group metals for the preferential oxidation of carbon monoxide. Angew Chem. Int. Ed. 51, 2920–2924. 10.1002/anie.201106702 22307960

[B10] Lu J. Aydin C. Browning N. Gates B. (2012). Imaging isolated gold atom catalytic sites in zeolite NaY. Angew Chem. Int. Ed. 51, 5842–5846. 10.1002/anie.201107391 22411626

[B11] Nieva Lobos M. Sieben J. Comignani V. Duarte M. Volpe M. Moyano E. (2016). Biochar from pyrolysis of cellulose: an alternative catalyst support for the electro-oxidation of methanol. Int. J. Hydrogen Energ. 41, 10695–10706. 10.1016/j.ijhydene.2016.04.041

[B12] Ormsby R. Kastner J. Miller J. (2012). Hemicellulose hydrolysis using solid acid catalysts generated from biochar. Catal. Today 190, 89–97. 10.1016/j.cattod.2012.02.050

[B13] Qiao B. Wang A. Yang X. Allard L. Jiang Z. Cui Y.. (2011). Single-atom catalysis of CO oxidation using Pt_1_/FeOx. Nat. Chem. 3, 634–641. 10.1038/nchem.1095 21778984

[B14] Seehra M. Ghosh B. (1988). Free radicals, kinetics and phase changes in the pyrolysis of eight American coals. J. Anal. Appl. Pyrol. 13, 209–220 10.1016/0165-2370(88)80023-8

[B15] Shen Y. Yoshikawa K. (2014). Tar Conversion and Vapor Upgrading via in Situ Catalysis Using Silica-Based Nickel Nanoparticles Embedded in Rice Husk Char for Biomass Pyrolysis/Gasification. Industrial and Engineering Chemistry Research. 53, 10929–10942. 10.1021/ie501843y

[B16] Shi Y. Zhao C. Wei H. Guo J. Liang S. Wang A.. (2014). Single-atom catalysis in mesoporous photovoltaics: the principle of utility maximization. Adv. Mater. 26, 8147–8153. 10.1002/adma.201402978 25312028

[B17] Wang H. Wang Q. Cheng Y. Li K. Yao Y. Zhang Q.. (2012). Doping monolayer graphene with single atom substitutions. Nano Lett. 12, 141–144. 10.1021/nl2031629 22136503

[B18] Wang J. Zhang uinian H. Wang ongwei C. Zhang an Y. Wang J. Zhao H. (2018). Co-synthesis of atomic Fe and few-layer graphene towards superior ORR electrocatalyst. Energ. Stor. Mater. 12, 1–7. 10.1016/j.ensm.2017.11.004

[B19] Wang L. Zhang S. Zhu Y. Patlolla A. Shan J. Yoshida H. (2013). Catalysis and *in situ* studies of Rh_1_/Co_3_O_4_ nanorods in reduction of NO with H_2_. ACS Catal. 3, 1011–1019. 10.1021/cs300816u

[B20] Wang S. Wang H. Yin Q. Zhu L. Yin S. (2014). Methanation of bio-syngas over a biochar supported catalyst. New J. Chem. 38:4471 10.1039/C4NJ00780H

[B21] Wu J. Liu Q. Wang R. He W. Shi L. Guo X. (2017). Coke formation during thermal reaction of tar from pyrolysis of a subbituminous coal. Fuel Process. Technol. 155, 68–73. 10.1016/j.fuproc.2016.03.022

[B22] Xiang C. Liu Q. Shi L. Liu Z. (2020). A study on the new type of radicals in corncob derived biochars. Fuel 277:118163 10.1016/j.fuel.2020.118163

[B23] Yan H. Cheng H. Yi H. Lin Y. Yao T. Wang C.. (2015). Single-atom Pd(1)/graphene catalyst achieved by atomic layer deposition: remarkable performance in selective hydrogenation of 1,3-butadiene. J. Am. Chem. Soc. 137, 10484–10487. 10.1021/jacs.5b06485 26268551

[B24] Yang X. Wang A. Qiao B. Li J. Liu J. Zhang T. (2013). Single-atom catalysts: a new frontier in heterogeneous catalysis. Accounts Chem. Res. 46, 1740–1748. 10.1021/ar300361m 23815772

[B25] Zhang H. Wei J. Dong J. Liu G. Shi L. An P.. (2016). Efficient visible-light-driven carbon dioxide reduction by a single-atom implanted metal-organic framework. Angew Chem. Int. Ed. 55, 14310–14314. 10.1002/anie.201608597 27736031

