# Supplementary material for: Radical-Assisted Formation of Pd Single Atoms or Nanoclusters on Biochar
Source: Front Chem. 2020 Nov 30;8:598352. doi: 10.3389/fchem.2020.598352 (PMC7734141; doi:10.3389/fchem.2020.598352)
Supplement: Supplementary file 1 [file Table_1.DOCX]

Radical-assisted formation of Pd single atoms or nanoclusters on biochar

Chong Xiang, Qingya Liu, Lei Shi, Zhenyu Liu^*^

State Key Laboratory of Chemical Resource Engineering, Beijing University of Chemical Technology, Beijing, 100029, China

**Supporting Information**

**Characterization of walnut shell and walnut shell derived chars**

**Sample preparation**

The walnut shell was ground and sieved to 0.106–0.250 mm size range and dried at 110 °C in a vacuum for 4 h.

**Char preparation**

A quartz crucible with 1 g walnut shell sample was placed in the center of a corundum tube of 1 m in length and 3.2 cm in inner diameter. The tube was heated in a furnace from the room temperature to the final temperature (T_f_) of 600 and 850 °C at a rate of 5 °C/min, and then kept at the T_f_ for 15 min before been cooled to the room temperature. The sample was purged with Ar of 0.99999 purity at a rate of 100 mL/min during the whole process. The char was discharged from the pyrolysis tube and sieved to smaller than 0.25 mm under an N_2_ atmosphere in a glovebox to yield the F-Char. The F-Char is termed F-Char-T_f_, such as F-Char-600. Some of the F-Char-T_f_ samples were fully exposed to O_2_ at the ambient temperature to yield O-Char-T_f_ and then purged by N_2_ at the ambient temperature to yield N-O-Char-T_f_.

**Ultimate and proximate analyses**

The ultimate analysis was performed on an elemental analyzer (Elementar Vario EL Cube) to quantify the samples’ C, H, N and S contents. These elements were converted to CO_2_, H_2_O, N_2_ and SO_2_, respectively, and then analyzed by a TCD detector, calibrated by sulfadiazine, with absolute errors of less than 0.1%.

The proximate analysis was performed on a thermogravimetric analyzer (TG, Setsys Evolution 24, Setaram) with approximately 15 mg sample in an argon flow of 100 mL/min and with a heating rate of 10 °C/min. The temperature was from room temperature to 900 °C, with a 30 min stay at 110 °C for moisture removal and a 20 min stay at 900 °C. The mass losses recorded at 110 °C and between 110-900 °C are the contents of moisture and volatiles, respectively. After staying at 900 °C for 20 min the char was combusted in a flow of 10% oxygen and balance argon at a rate of 100 mL/min for 1 h to yield the contents of fixed carbon and ash.

The ultimate and proximate analyses data of the walnut shell and two chars are shown in Table S1.

**Table S1.** Ultimate and proximate analyses of walnut shell, N-O-Char-600 and N-O-Char-850

| Sample | Ultimate analysis (wt.%, daf) | | | | | | Proximate analysis (wt.%) | | |
| --- | --- | --- | --- | --- | --- | --- | --- | --- | --- |
|  | C | H | N | O* | S | H/C^α^ | M_ad_ | A_d_ | V_daf_ |
| Walnut shell | 51.8 | 6.8 | 0.1 | 39.7 | 0.0 | 1.58 | 3.7 | 3.9 | 79.5 |
| N-O-Char-600 | 85.3 | 1.7 | 0.1 | 13.0 | 0.0 | 0.24 | 3.5 | 5.7 | 26.7 |
| N-O-Char-850 | 94.5 | 1.0 | 0.1 | 4.4 | 0.0 | 0.13 | 0.5 | 8.6 | 4.5 |

ad: air-dry basis; d: dry basis; daf: dry-and-ash-free basis; M: moisture; A: ash; V: volatile matter content; *: by difference; ^α^: atomic ratio.

**Impregnation of Pd on chars**

The chars were impregnated with a solution containing palladium acetate and toluene (termed as Pd(Ac)_2_/toluene) or tetrahydrofuran (THF, termed as Pd(Ac)_2_/THF) at a solvent to char ratio of 0.3 (mL/g). The impregnation of F-Char-600 and F-Char-850 were carried out in a glovebox under nitrogen while that of N-O-Char-600 and N-O-Char-850 were carried out in a parafilm covered beaker in air. The samples were then dried under vacuum at 110 °C for 4 h.

**Electron spin resonance (ESR) analysis**

Approximately 3 mg F-Char-T_f_ was loaded into an ESR sample tube of 2 mm in diameter and 30 mm in length in the glovebox under a N_2_ atmosphere. The sample tube was then sealed with parafilm to keep it from the ambient environment and subjected to the ESR analysis. After the ESR analysis, the sample was purged with flowing O_2_ through a syringe for 5, 10, 20, 30, 60, 120, 180, 300 or 600 s to yield O-Char-T_f_, and then subjected to the ESR analysis again. After this operation, the O-Char-T_f_ sample was purged with flowing N_2_ through a syringe for 5, 10, 20, 30, 60, 120, 180, 300 or 600 s to yield N-O-Char-T_f_, and then also subjected to the ESR analysis. This O_2_-and-then-N_2_ purging cycle was repeated three times for each char sample.

The ESR used was e-scan (Bruker, Switzerland) operated at 9.5 GHz and 1.578 mW under a central magnetic field of 3485 G, with a modulation amplitude of 1.0 G, a sweep width of 100 G, sweep time of 20.97 s, and a time constant of 0.041 s. The radical signal was calibrated by DPPH (1,1-Diphenyl-2-picrylhydrazyl, purity greater than 98%). The calibrated radical signal was converted to radical concentration *C*_R_ by Eqs. (1a) and (1b), where *N*_R, Sample_ and *N*_R, DPPH_ are quantities of radicals in the sample and DPPH, respectively, A_sample_ and A_DPPH_ are ESR peak areas of sample and DPPH, respectively, while m_sample_ is the sample mass (g).

*C*_R_ = *N*_R, sample_/m_sample_ (1a)

*N*_R, sample_/A_sample_ = *N*_R, DPPH_/A_DPPH_ (1b)

The sample tube showed little influence on ESR signal. At least 2 ESR measurements were carried out for each sample and the experimental errors were less than 5%.

**High angle annular dark field aberration-corrected scanning transmission electron microscope (HAADF-STEM) analysis**

After immersing for a certain period of time, the sample is washed with ethanol and filtered 3 times, and the sample remaining on the filter paper is dried in a blast dryer at 110 °C for 4 hours before HAADF-STEM (Themis) analysis. HAADF-STEM with a molybdenum mesh microgrid ultra-thin carbon film was used to characterize the Pd size distribution on the chars. The samples were ultrasonically dispersed in ethanol for the measurement.

**Inductively coupled plasma atomic emission spectroscopy (ICP-AES) analysis**

The Pd supported chars were subjected to ICP-AES analysis to determine their Pd contents. The ICP-AES was Icap 6000 SERIES (Thermo Scientific). The samples were digested in the equal mass HNO_3_ (68%) for 48 h and then washed by deionized water. The solution was analyzed for Pd content and the result was converted to the char basis. The final results are listed in Table S2.

Table S2. Pd loading of various chars by ICP

| Sample | Pd (wt.%) |
| --- | --- |
| F-Char-600+Pd/toluene 24 h | 0.017 |
| N-O-Char-600+Pd/toluene 24 h | 0.024 |
| F-Char-600+Pd/toluene 84 h | 0.061 |
| N-O-Char-600+Pd/toluene 84 h | 0.075 |
| N-O-Char-600 84 h | 0.001 |
