# Supplementary figures and images for: Radical-Assisted Formation of Pd Single Atoms or Nanoclusters on Biochar
Source: Front Chem. 2020 Nov 30;8:598352. doi: 10.3389/fchem.2020.598352 (PMC7734141; doi:10.3389/fchem.2020.598352)

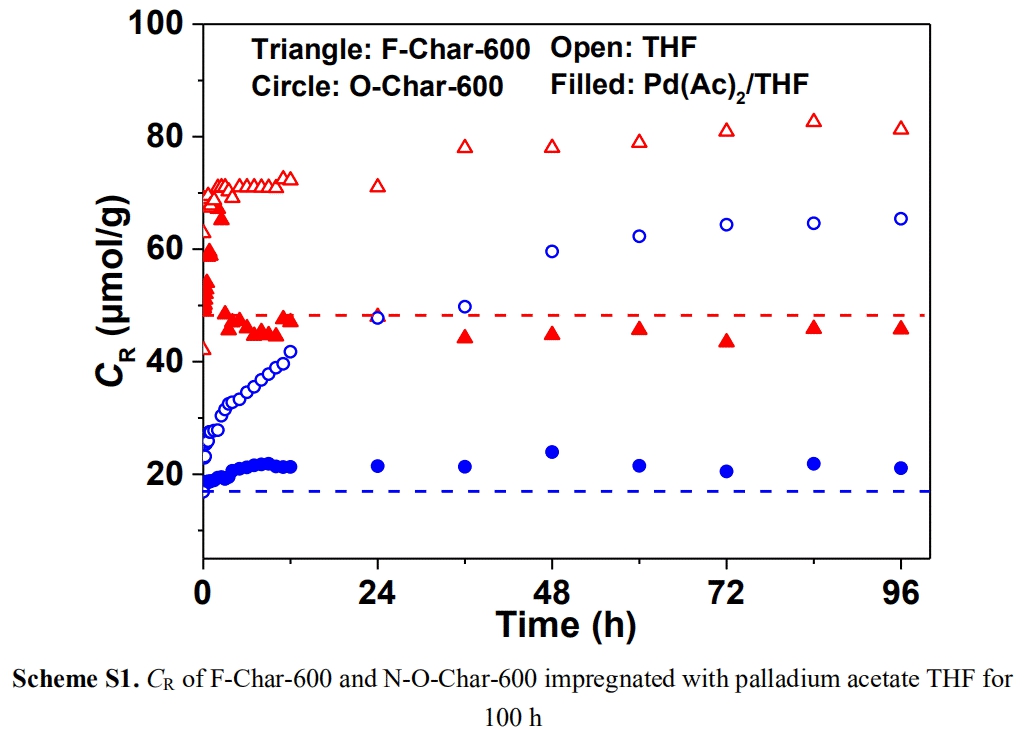

Supplement: Supplementary file 2 [file Scheme_1.JPEG]

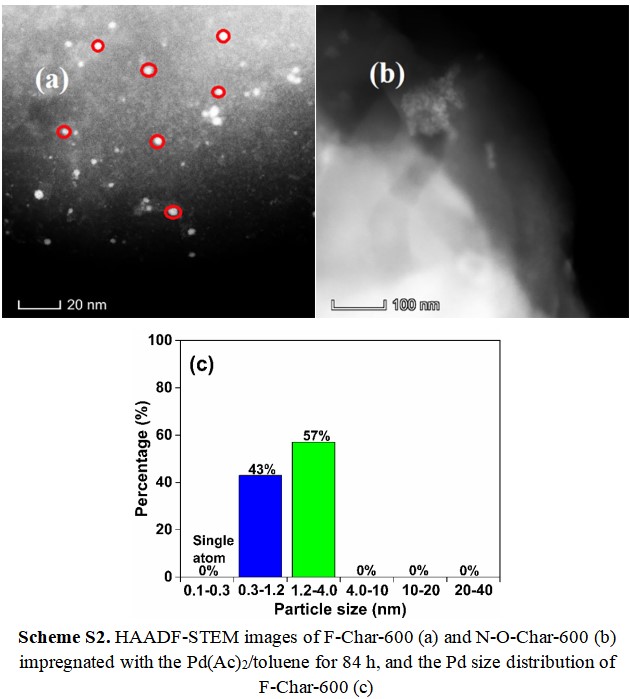

Supplement: Supplementary file 3 [file Scheme_2.JPEG]

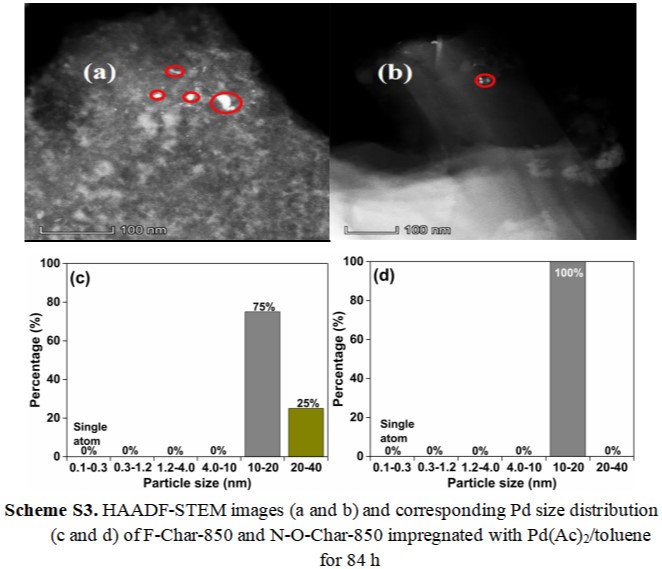

Supplement: Supplementary file 4 [file Scheme_3.JPEG]
